# Current and future therapies for type 1 diabetes

**DOI:** 10.1007/s00125-021-05398-3

**Published:** 2021-02-17

**Authors:** Bernt Johan von Scholten, Frederik F. Kreiner, Stephen C. L. Gough, Matthias von Herrath

**Affiliations:** 1 Global Chief Medical Office, Novo Nordisk A/S, Søborg, Denmark; 2 Type 1 Diabetes Center, The La Jolla Institute for Immunology, La Jolla, CA USA

**Keywords:** Adjunctive therapies, Beta cell preservation, Immunomodulation, Prevention, Review, Type 1 diabetes

## Abstract

In type 1 diabetes, insulin remains the mature therapeutic cornerstone; yet, the increasing number of individuals developing type 1 diabetes (predominantly children and adolescents) still face severe complications. Fortunately, our understanding of type 1 diabetes is continuously being refined, allowing for refocused development of novel prevention and management strategies. Hitherto, attempts based on immune suppression and modulation have been only partly successful in preventing the key pathophysiological feature in type 1 diabetes: the immune-mediated derangement or destruction of beta cells in the pancreatic islets of Langerhans, leading to low or absent insulin secretion and chronic hyperglycaemia. Evidence now warrants a focus on the beta cell itself and how to avoid its dysfunction, which is putatively caused by cytokine-driven inflammation and other stress factors, leading to low insulin-secretory capacity, autoantigen presentation and immune-mediated destruction. Correspondingly, beta cell rescue strategies are being pursued, which include antigen vaccination using, for example, oral insulin or peptides, as well as agents with suggested benefits on beta cell stress, such as verapamil and glucagon-like peptide-1 receptor agonists. Whilst autoimmune-focused prevention approaches are central in type 1 diabetes and will be a requirement in the advent of stem cell-based replacement therapies, managing the primarily cardiometabolic complications of established type 1 diabetes is equally essential. In this review, we outline selected recent and suggested future attempts to address the evolving profile of the person with type 1 diabetes.

**Supplementary Information:**

The online version contains a slide of the figure for download available at 10.1007/s00125-021-05398-3.

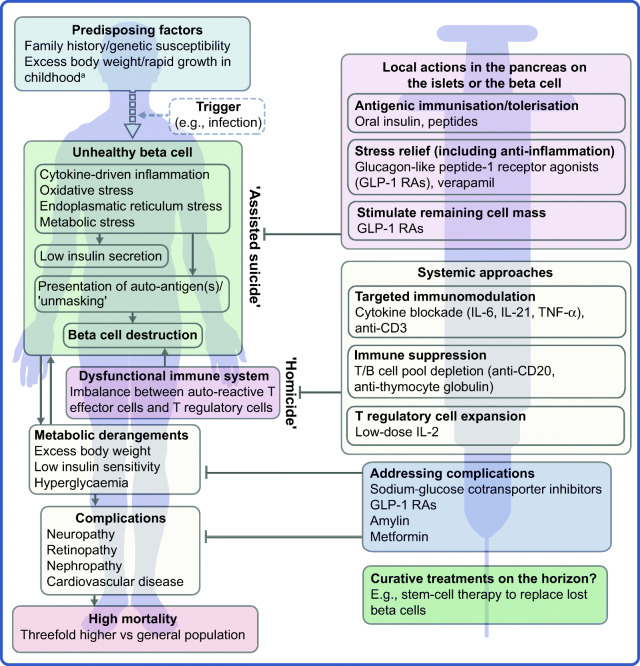

## Introduction

In addition to prolonging the life expectancy of people living with type 1 diabetes, the discovery of insulin a century ago revolutionised the management of this chronic autoimmune disease. Today, type 1 diabetes is the most common type of diabetes in children, and estimates suggest that around 100,000 children develop the disease every year [1]. Unfortunately, despite the availability of advanced insulins, affected individuals remain at high risk of serious complications, including cardiovascular mortality [2–4]. New interventions are, therefore, urgently required to improve the prognosis for the increasing number of people who are diagnosed with type 1 diabetes each year.

**Fig. 1:**
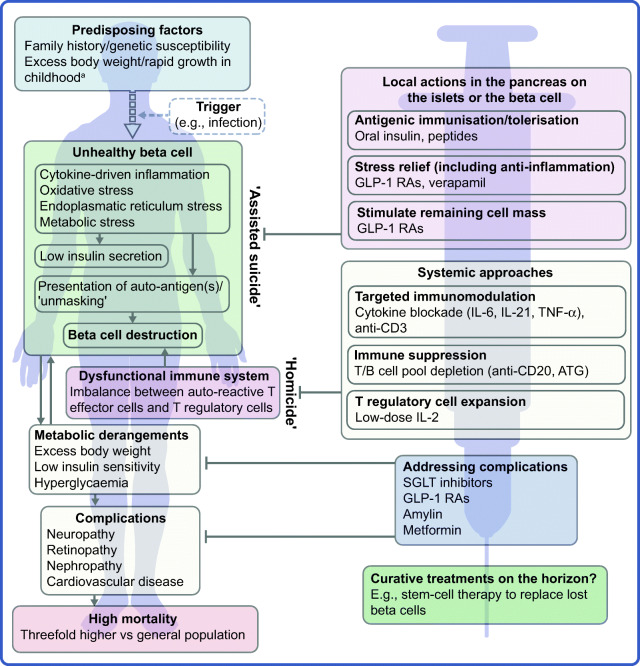
Hallmarks of the evolving profile of the individual with type 1 diabetes, and current and future options for the prevention of this disease and for the management of its associated complications. ^a^According to some recent evidence [124–130]. This figure is available as a downloadable slide

The profile of the person with type 1 diabetes is evolving and, with that, our understanding of the disease. The overall pathophysiological feature is loss of functional beta cell mass in the pancreatic islets of Langerhans (Fig. 1) [5]. Hypotheses suggest that the loss of functional beta cell mass occurs in a chain of events analogous to an ‘assisted suicide’ [6, 7], where the demise of the beta cell is likely due to a combination of a dysfunctional beta cell that becomes more visible to the immune system, which, in turn, overreacts and destroys the beta cell.

In its early stage (Stage 1), type 1 diabetes is usually asymptomatic; however, the development of autoimmunity is often detectable in early life, with circulating autoantibodies targeting insulin or other proteins, such as GAD65, insulinoma-associated protein 2 (IA­2) or zinc transporter 8 (ZNT8) [5]. When a large portion of the beta cell mass has become dysfunctional or lost, asymptomatic dysglycaemia (Stage 2) and, later, symptoms of hyperglycaemia (Stage 3) ensue due to insufficient or absent insulin secretion.

Type 1 diabetes is a polygenic disorder, in which susceptibility loci or genetic variation contributes to disease risk. The HLA region on chromosome 6 is the main susceptibility locus and, in recent years, many other loci across the genome have been associated with an increasing risk of the disease [8]. However, from studies in monozygotic twins, for whom the onset of type 1 diabetes can vary considerably [9], it has become evident that non-genetic factors play a major role in triggering or perpetuating overt type 1 diabetes. A multitude of efforts have failed at robustly identifying such factors, strongly indicating that no single pathogen is responsible. Viral infections have been suggested, including enteroviruses and human herpesvirus-6 [10–13]. Of note, however, studies (mainly in animals) have also suggested that several viral infections may prevent the development of type 1 diabetes [14, 15], in line with the ‘hygiene hypothesis’ [16, 17].

People living with type 1 diabetes remain dependent on exogenous insulins as the cornerstone therapeutic option [18]. Since the isolation of insulin in 1921, novel and versatile formulations, analogues and delivery vehicles have been introduced [19, 20]. Together with much improved glucose monitoring, these advances have contributed to the increases in the survival and life expectancy of individuals with type 1 diabetes [21]. Still, only a minority of people with type 1 diabetes achieve recommended glycaemic and time-in-range targets [22], and hyperglycaemia continues to be a risk factor for short-term metabolic and long-term macro- and microvascular complications [2, 23–25]. Further, the use of exogenous insulins requires unremitting glycaemic monitoring and dose titration to mitigate the risk of hypoglycaemia. The all-cause mortality risk is around threefold higher for the individual with type 1 diabetes than for the general population [2–4, 26], and type 1 diabetes has been shown to be linked to cardiovascular outcomes more than any other disease, including type 2 diabetes [2].

As mentioned earlier, novel interventions are needed for the prevention and management of type 1 diabetes. Whilst progress has been limited, the evolving profile of a person with type 1 diabetes suggests that beyond ensuring accurate titration of exogenous insulin, efficient management of the disease should rely on other additional principles. First, there is an obvious need to act early to prevent or delay the destruction of functional beta cell mass by immunomodulatory intervention or other disease-modifying means. Second, stimulating or reprogramming the remaining beta cell mass to secrete insulin in a balanced way is required to avoid major blood glucose excursions with the lowest possible exogenous insulin dose. Third, reducing the risk of long-term complications, such as cardiovascular and renal outcomes, seems increasingly important (Fig. 1). Below we review selected current and in-development interventions meeting these three criteria (Table 1).

**Table 1:** Non-insulin agents for the prevention and management of type 1 diabetes

Mechanism of action/target	Agent	Reference of selected main studies or ClinicalTrials.gov registration no.
Systemic approaches		
T effector cells	Teplizumab (anti-CD3)	[27–29, 31]; NCT03875729
	Otelixizumab (anti-CD3)	[30]
	ATG	[36]
	Abatacept (anti-CD80 and anti-CD86)	[42, 43]
	Alefacept	[39]
	Anti-IL-21 antibody	[55, 56, 59, 61]
B cells	Rituximab (anti-CD20)	[34, 35]
T regulatory cell expansion	Low-dose IL-2	[44–48]
Anti-inflammation	Infliximab, adalimumab, etanercept, golimumab (anti-TNF-α)	[49–51]
	Tocilizumab (anti-IL-6R)	[53, 54]; NCT02293837
	GLP-1 RAs	[120–123]
Islet/beta cell-specific approaches		
Islet-antigen tolerisation/immunisation	Oral insulin	[63–66]
	GAD65	[67]
	Peptides	[68, 69]
Beta cell stress relief and stimulation	GLP-1 RAs	[91, 92, 113–117, 135–138]
	Verapamil	[102, 103]
Cardiometabolic improvements^a^		
SGLT inhibition	Dapagliflozin, empagliflozin, sotagliflozin	[78–83, 88, 89]
GLP-1 agonism	Exenatide, liraglutide, dulaglutide, semaglutide	[90–92, 135–138]
Other/unspecific	Amylin (pramlintide)	[145, 146]
	Metformin	[73–76]

^a^Including blood glucose levels, body weight, blood lipids, blood pressure and cardiorenal risk

## Immune-focused therapies

The overarching goal of immune-focused therapies in type 1 diabetes is to prevent or delay the loss of functional beta cell mass. The traditional understanding of autoimmunity in type 1 diabetes has focused on systemic immune dysregulation and on autoreactive T cells that have evaded thymic selection and migrated to the periphery, where they destroy islets. This view on the pathogenesis of type 1 diabetes has been referred to as T cell-mediated ‘homicide’ [6]. Thus, recent efforts have concentrated on cell- or cytokine-directed interventions, which have been successful in other autoimmune diseases. Targeting T cells or proinflammatory cytokines remain valid efforts and many agents are in active development; so far, however, these approaches have been only partly successful. This arguably indicates a need to refocus hypotheses, as discussed later in this review (see ‘Future perspectives’ section), where we outline how the beta cell itself contributes to its own demise (the ‘assisted suicide’ hypothesis).

### Cell-directed interventions

In line with the traditional immune-centric view on the pathogenesis of type 1 diabetes, many immunomodulatory strategies have focused on antibodies targeting T effector cells. The anti-CD3 antibodies teplizumab and otelixizumab have shown some attenuation of loss of beta cell function [27–30]. A Phase II trial with relatives with a high risk of developing type 1 diabetes indicated a more than 50% risk reduction with teplizumab (HR 0.41 vs placebo) and clinical type 1 diabetes diagnosis was delayed by 1.5–2 years [31]. Accordingly, teplizumab has recently been granted a breakthrough therapy status by the US Food and Drug Administration. An ongoing Phase III trial (PROTECT; ClinicalTrials.gov registration no. NCT03875729) aims to evaluate the benefits and safety of teplizumab in children and adolescents with recently diagnosed type 1 diabetes.

The presence of autoantibodies against beta cell antigens, such as GAD65 and insulin, has spurred attempts targeting B cell-related molecules. These efforts have been somewhat successful in animal models [32, 33], as well as clinically, most prominently with the B cell-depleting anti-CD20 antibody rituximab. Although rituximab led to detectable protraction of beta cell function [34], the effect was transient [35], exemplifying the fact that B cell-directed therapy alone does not appear to sustainably prevent or ameliorate beta cell autoimmunity. So far, however, B cell-directed agents have not been tested in the early disease stage, precluding conclusions regarding the usefulness of such interventions in delaying or even preventing progression to later stages.

In clinical investigations, low-dose anti-thymocyte globulin (ATG) treatment significantly (vs placebo) preserved C-peptide secretion and improved glycaemic control in children, as well as adults, with new-onset type 1 diabetes [36–38]. The potential benefits of ATG appear to depend on the dose level and the age of the recipients, and the clinical utility of the approach remains to be established. ATG in combination with granulocyte colony stimulating factor (GCSF) was also explored based on the hypothesis of a synergistic benefit of the combination of transient T cell depletion via low-dose ATG with the upregulation of activated T regulatory cells and tolerogenic dendritic cells induced by GCSF. However, the combination did not appear to offer a synergistic effect; in contrast to the use of ATG alone, ATG plus GCSF did not appear to be better than placebo in preserving C-peptide secretion [37].

Tissue-resident memory T effector cells, which likely play a role in many organ-specific autoimmune diseases, such as type 1 diabetes, are very difficult to eliminate. Alefacept, a T cell-depleting fusion protein that targets CD2 and, therefore, memory T effector cells, was tested in adolescents and young adults with Stage 3 type 1 diabetes in the T1DAL trial [39]. Although the trial did not complete enrolment as planned, it reported a trend for benefits with regard to beta cell preservation, reduced insulin requirements and low risk of hypoglycaemia that persisted throughout the follow-up of 15 months after treatment.

Importantly, whether considering the targeting of the T or B cell in type 1 diabetes, sufficient long-term benefits via systemic cell pool depletion comes with an inherent risk of introducing equally long-term or even irreversible changes to the immune system. Such changes may predispose the patient to a less favourable prognosis for chronic viral infections. For example, reactivation of Epstein-Barr virus (EBV) has been observed after anti-CD3 therapies [40, 41]. Mitigating such risks may be achieved using carefully tailored dosing regimens and monitoring; still, the seriousness of the risks may indicate an unfavourable benefit:risks balance. Therefore, non-depleting immunomodulation has been explored. For example, 24-month blockade of CD80 and CD86 via the cytotoxic T-lymphocyte-associated protein 4 (CTLA-4)-immunoglobulin fusion molecule abatacept markedly prolonged beta cell function in new-onset type 1 diabetes and was accompanied by increased numbers of naive T cells [42, 43].

### Cytokine-directed interventions

Anti-inflammatory cytokine-specific compounds, which are successfully used, for example, in rheumatic diseases, have been tested as alternatives to directly targeting the T or B cell in type 1 diabetes, as briefly summarised below. In addition, to stimulate an increase in T regulatory cells, low-dose IL-2 treatment has also been tested and the results have been somewhat promising [44–48], with recent developments mitigating earlier caveats, which included an arguably narrow dose range and lack of full specificity for T regulatory cells.

Blockade or antagonism of the central proinflammatory cytokine TNF-α using infliximab, adalimumab or the receptor fusion protein etanercept have shown some potential in type 1 diabetes, with indications of improved glycaemic control and C-peptide secretion [49, 50]. More recently, a C-peptide-sparing effect of TNF-α blockade was reported with golimumab use, after 1 year in children and young adults with type 1 diabetes [51].

IL-6 is another proinflammatory cytokine that has been targeted with success in multiple other autoimmune diseases [52]. Although its role in type 1 diabetes is not established, IL-6 has been suggested as a target [53]. Of note, IL-6 has been shown to protect the beta cell from oxidative stress and is constitutively expressed by pancreatic alpha and beta cells, indicating important physiological roles [54]. In type 1 diabetes, the EXTEND Phase II trial of tocilizumab, a monoclonal antibody against the IL-6 receptor, was recently completed (ClinicalTrials.gov registration no. NCT02293837).

IL-21 has been proposed as an attractive target in type 1 diabetes [55, 56]. Physiologically, IL-21 is important not only for the function of T helper (Th) cells (Th17 and T follicular helper cells) but also for the generation and migration of CD8^+^ T cells. CD8^+^ T cells are now considered the chief T cell type accumulating in and around islets [57, 58] with pre-proinsulin emerging as a pivotal autoantigen driving their infiltration in type 1 diabetes [59]. IL-21 neutralisation has been shown to prevent diabetes in mice [60], and a C-peptide-sparing benefit of anti-IL-21 alone or in combination with the glucagon-like peptide-1 (GLP-1) receptor agonist (RA) liraglutide has been observed in a clinical proof-of-concept study [61], as described further below. Reassuringly, non-clinical models, including a viral type 1 diabetes model, showed a minor impact of IL-21 blockade on the immune repertoire [55].

### Antigen vaccination

With the appeal of having no expected effect on acquired immunity, the overall aim of beta cell antigen vaccination is to induce tolerance by balancing the T cell population between auto-aggressive T effector cells and autoantigen-specific T regulatory cells. Induction of T regulatory cells carries the potential benefit of also downregulating the activity of proinflammatory antigen-presenting cells. The topic has been extensively reviewed in the past [62]. Briefly, inspired by successes with vaccination against, for example, peanut allergy, tolerisation of T effector cells has been attempted using administration of whole antigens, such as oral insulin, or of peptides. Whilst the concepts are promising and under active investigation, their effectiveness in humans is yet to be proven. For example, in at-risk children, oral insulin administration has previously failed to prevent type 1 diabetes [63, 64], speculatively due to a suboptimal dose level or unclear effects across risk-specific subgroups [65, 66], including those defined by insulin gene polymorphisms. Similar results and considerations have been reported for immunisation with GAD65 [67] and for peptide-based therapies [68, 69]. Further, the lack of full clarity regarding the mechanisms at play with antigen-based therapies outlines a number of shortcomings, including the fact that no biomarker is currently available to assist in establishing the optimal dose regimen.

## Non-immunomodulatory adjunctives

We next focus on selected compounds that have gained attention due to their potential benefits as adjuncts to insulin in type 1 diabetes.

### Amylin

Amylin deficiency is a recognised feature of type 1 diabetes [70]. As a neuroendocrine hormone, amylin inhibits glucagon secretion and contributes to reducing postprandial glucose variability. As an adjunct to meal-time insulin, the injectable amylin analogue pramlintide is approved only in the USA for the treatment of type 1 and type 2 diabetes alike [71]. In type 1 diabetes, pramlintide has been shown to improve postprandial glucose levels to some extent [72]. Its clinical use has been limited, arguably because of the modest efficacy alongside the occurrence of side effects, such as nausea and, most importantly, postprandial hypoglycaemia.

### Metformin

Metformin is a low-cost agent with glucose-lowering effects that mainly occur via decreased hepatic glucose production. It is not a guideline-recommended option in type 1 diabetes. However, partly because of its ameliorating effect on insulin resistance, metformin has been somewhat promising in managing the disease, especially in children and adolescents, as well as in obese people with type 1 diabetes, with studies indicating reduced insulin requirements and body weight reduction [73–75]. In the large REducing With MetfOrmin Vascular Adverse Lesions (REMOVAL) trial, however, metformin did not reduce the long-term insulin needs or improve glycaemic control in people with long-standing type 1 diabetes and multiple cardiovascular risk factors [76].

### Sodium-glucose cotransporter inhibitors

Sodium-glucose cotransporter (SGLT) inhibitors lower blood glucose levels by restraining the absorption of glucose in the small intestine and promoting the renal excretion of glucose [77]. Results with dapagliflozin, empagliflozin and sotagliflozin have indicated benefits of SGLT inhibition in managing type 1 diabetes when added to insulin [78–83]. Significant benefits included reduced insulin dose requirements, improved glycaemic control and reduced body weight [84]. So far, sotagliflozin and dapagliflozin are approved in Europe and Japan (but not the USA) as adjuncts to insulin for the management of overweight or obese people with type 1 diabetes when optimally titrated insulin alone does not provide adequate glycaemic control. Importantly, however, data suggest that the use of SGLT inhibitors in type 1 diabetes is associated with markedly increased risk of diabetic ketoacidosis [85–87]; for sotagliflozin, a 5–17-fold risk increase was noted [88]. These observations prompted the formation of an international consensus on recommendations for the use of SGLT inhibition in type 1 diabetes [89] as well as a suggestion that treatment should be overseen by specialists [88].

### GLP-1 RAs

GLP-1 is a hormone of the incretin system that is secreted upon food intake. A marked uptake has been seen in the use of GLP-1 RAs in type 2 diabetes due to their pleiotropic glucose-dependent effects that improve glycaemic control and reduce body weight [90]. In contrast, GLP-1 agonism for the treatment of type 1 diabetes remains unproven, with initial results from smaller investigator-conceived studies being inconclusive. Recently, Phase II findings with the short-acting GLP-1 RA exenatide in adults with type 1 diabetes were negative. In two larger Phase III trials (ADJUNCT ONE and ADJUNCT TWO), the GLP-1 analogue liraglutide used as an adjunct to insulin appeared well-tolerated and improved HbA_1c_ and reduced body weight [91, 92]. Both ADJUNCT trials indicated a minor increase in the risk of hypoglycaemia and hyperglycaemia with ketosis with liraglutide use, whereas the risk of diabetic ketoacidosis was negligible. Subsequently, a plethora of investigations have reached similar conclusions [93–101]. Nonetheless, the use of GLP-1 RAs in type 1 diabetes remains potentially useful, as discussed below.

### Verapamil

Verapamil is a common calcium-channel blocker used for decades as an anti-hypertensive agent. In mouse models of type 1 diabetes, verapamil promoted survival of functional beta cells via a mechanism that involves reduced expression of the cellular redox regulator thioredoxin-interacting protein [102]. In a smaller Phase II trial, verapamil was better than placebo for preserving meal-stimulated C-peptide secretion in adults with type 1 diabetes and no safety concerns were identified [103]. Despite these findings, however, the place for verapamil as a disease-modifying agent in type 1 diabetes remains to be fully established.

## Future perspectives

Although research into type 1 diabetes prevention and disease modification continues to produce encouraging data, none of the approaches discussed above appears sufficiently effective alone in preventing or managing type 1 diabetes. Future endeavours will, therefore, require a novel focus, leveraging prior experience with regard to the immunopathophysiology of type 1 diabetes, whilst also exploring the promise of combination therapies that integrate tried or new treatment modalities. In addition, lessons learned from type 2 diabetes with regard to the beneficial effects of certain agents on, for example, body weight and cardiorenal risk may also prove relevant in type 1 diabetes. We review selected future prospects addressing these aspects below.

Of further note, the lack of sufficient efficacy of previously tested therapies may also be related to the fact that type 1 diabetes is a heterogenous disease with diverse disease stages (Stages 1 to 3) and modifiers, such as age of onset or clinical diagnosis. Identifying the optimal timing of each type of intervention relative to the disease stages and the age of the patient is, therefore, important. For example, initiating an immunomodulatory intervention at Stage 1 (i.e. prior to clinical diagnosis) is not a straightforward decision and may be associated with clinical inertia. Moreover, an increased focus on disease endotypes (i.e. different biological processes under the type 1 diabetes umbrella) was recently suggested to ensure a precision-medicine approach to type 1 diabetes research and management [104].

### Immune interventions

It is becoming increasingly clear that autoreactivity to islet antigens is also present in healthy individuals [59] and autoimmunity recurs after autologous nonmyeloablative haematopoietic stem cell transplantation [105, 106]. Thus, in line with the ‘assisted suicide’ theory introduced earlier [6, 7], it is also increasingly apparent that the development of type 1 diabetes does not only involve dysfunctional islets, but also beta cells that ‘unmask’ themselves to immune recognition and destruction. This notion supports two central realisations; first, it might explain why, in previous studies, immune therapy alone has failed to protect beta cell function over longer periods of time after onset of diabetes. Second, looking forward, novel type 1 diabetes therapies should pursue the holy grail of type 1 diabetes immune therapy: essentially agents that act locally in the islets, within the pancreas, either targeting the immune cells destroying the beta cell or the beta cell itself. Knowledge gained over the years regarding the beta cell has suggested multiple, yet putative reasons for the ‘unmasking’ of these cells. Potential reasons include the facts that beta cells are especially biosynthetically active and systemically exposed [107] and, therefore, susceptible to stress-induced production of autoantigenic proteins during, for example, infections [108–110]. Moreover, the beta cell might be vulnerable to both cytokine-mediated destruction [111] and various types of endoplasmic reticulum stress [112]. Relieving the beta cell of these burdens may provide an opportunity to save the beta cell without resorting to aggressive immune suppression.

With this in mind, we see the following two promising avenues as deserving increased focus going forward: (1) therapies aimed at inducing tolerance to beta cell antigens; and (2) the use of GLP-1 RAs that directly target the beta cells to enhance their function whilst also protecting them from immune-mediated inflammatory stress.

As discussed above, achieving antigenic tolerance has, so far, proven elusive but carries the crucial potential of leaving the overall capacity of the immune system intact whilst suppressing only the diabetogenic cell populations. Future studies need to establish whether inducing tolerance in humans can be achieved by clonal anergy or clonal deletion of effector cells, or whether antigen-specific regulatory cells may be able to suppress autoreactivity locally. Moreover, it needs to be clarified to what extent tissue-resident memory effector cells can be eliminated.

Recent evidence from rodent models indicates a role for GLP-1 RAs in protecting beta cells from apoptosis and in promoting beta cell replication and mass [113–117]. As such, although this remains to be confirmed, it is conceivable that GLP-1 RAs may offer a way to prevent the ‘unmasking’ of the beta cell to immune effector cells, for example, by downregulating expression of MHC class I proteins. Intriguingly, unpublished non-clinical evidence shows that liraglutide also limits immune cell infiltration into pseudo-islets (M. von Herrath, unpublished results). In addition, studies in NOD mice have shown that GLP-1 RAs administered in combination with various immunomodulatory agents, including anti-CD3 compounds [118], were more efficient in inducing diabetes remission than when given as monotherapy [119]. Furthermore, the anti-inflammatory effects of GLP-1 RAs are well-documented, with liraglutide being associated with reduced systemic levels of C-reactive protein and of proinflammatory cytokines, such as TNF-α, IL-1β and IL-6 [120–123]. Whilst these findings have mainly been observed in animal models or in type 2 diabetes, their relevance to (clinical) type 1 diabetes is conceivable but, so far, largely unexplored.

### Management of cardiometabolic complications

A person diagnosed with type 1 diabetes faces a high risk of serious complications and of premature death, primarily for cardiovascular causes. This warrants a therapeutic focus on the broad pathophysiology of the disease.

Further, whilst the exact connections between excess body weight and type 1 diabetes remain debatable [124], the increased incidence of type 1 diabetes seems to coincide with the rapid rise in the prevalence of obesity [125, 126]. Recent evidence suggests that a high BMI may exacerbate the early-stage immune-mediated beta cell destruction in type 1 diabetes, especially in children and adolescents [127]. Evidence also points to an impact of rapid growth in early childhood [128], and a positive correlation between the age of type 1 diabetes onset and BMI has been observed [129]. The ‘accelerator hypothesis’ views high BMI and low insulin sensitivity as triggers for type 1 diabetes onset [130] and the term ‘double diabetes’ has been suggested to describe an amalgam of type 1 diabetes with parallel and separate pathophysiological processes typically associated with type 2 diabetes, such as obesity and insulin resistance [131].

Use of SGLT inhibitors or GLP-1 RAs as adjuncts to insulin admittedly holds promise in ameliorating multiple type 1 diabetes complications. For example, evidence suggests that SGLT inhibitors offer cardiorenal protection [132, 133], at least in type 2 diabetes, putatively owing to clinically unproven mechanisms of action beyond improved glucose homeostasis [134]. Moreover, a few GLP-1 RAs (dulaglutide, liraglutide and semaglutide) are now indicated to reduce cardiovascular risk in people with type 2 diabetes and established cardiovascular disease, and a protective effect of GLP-1 RAs on the kidneys is suggested from a range of cardiovascular outcome trials (CVOTs) in type 2 diabetes [135–138]. In addition, both SGLT inhibitors and GLP-1 RAs, especially second-generation GLP-1 RAs (e.g., semaglutide), are associated with a meaningful reducing effect on body weight.

### Combination therapies

Combination therapies that work via two mechanistically distinct targets to integrate immune modulation with a beta cell-specific component have been suggested [139–141] and encouraged [142]. Truly advantageous combination therapies are arguably those in which the components target different pathogenic pathways (for example, systemic vs beta cell-specific pathways), thereby synergising in terms of the beneficial effects. These combination therapies should also be safe and well-tolerated alone and in combination.

Known ongoing efforts are sparse but include the combination of ATG and GCSF (as discussed above) and the combination of targeted immune modulation via an anti-IL-21 antibody in combination with a GLP-1 analogue (liraglutide). In addition to the potential of preserving functional beta cell mass by leveraging the immunomodulatory and anti-inflammatory properties of both the anti-IL-21 antibody and liraglutide, their combination addresses the need to manage the symptoms and complications of established type 1 diabetes, as discussed earlier. As previously mentioned, results from a clinical proof-of-concept trial recently found that anti-IL-21 plus liraglutide was significantly better than placebo in preserving C-peptide secretion over a period of 54 weeks [61]. The benefits diminished after treatment cessation; however, the treatment appeared safe and well-tolerated.

### Stem cell replacement therapy

On the horizon, we approach the promise of stem cell-based therapies [143], offering a potential cure by replacing or supplementing beta cells that have been lost or have become dysfunctional. Stem cell-derived beta cells, however, also need to be rescued from immune-mediated destruction, suggesting that some degree of immunomodulation will be needed, even in the advent of viable stem cell therapy in type 1 diabetes, unless a fully effective immune-defying capsule is available [144]. In this context, better prevention or treatment regimens will also be useful for enabling longer-term beta cell graft acceptance.

## Closing thoughts

Whilst many intriguing non-insulin therapies have failed to fully meet their potential in the past few decades, hope remains that the knowledge gained has carved out paths towards better options for the prevention and management of type 1 diabetes. Taken together, in our view, stem cell replacement therapies and a refocused development of safe and well-tolerated combination therapies are the most promising emerging preventive or therapeutic avenues. In parallel, reinforced efforts to predict or diagnose type 1 diabetes as soon as possible are equally important in light of the fact that even the best interventions need to be introduced as early as possible to effectively preserve or rescue beta cells in individuals with this condition.

## Supplementary Information

Figure slide (PPTX 230 kb)

## Abbreviations

ATG: Anti-thymocyte globulin
GCSF: Granulocyte colony stimulating factor
GLP-1: Glucagon-like peptide-1
RA: Receptor agonist
SGLT: Sodium-glucose cotransporter
Th: T helper
