# Supplementary material for: Current and future therapies for type 1 diabetes
Source: Diabetologia. 2021 Feb 17;64(5):1037–48. doi: 10.1007/s00125-021-05398-3 (PMC8012324; doi:10.1007/s00125-021-05398-3)
Supplement: Supplementary file 1 — (PPTX 230 kb) [file 125_2021_5398_MOESM1_ESM.pptx]

## Slide 1
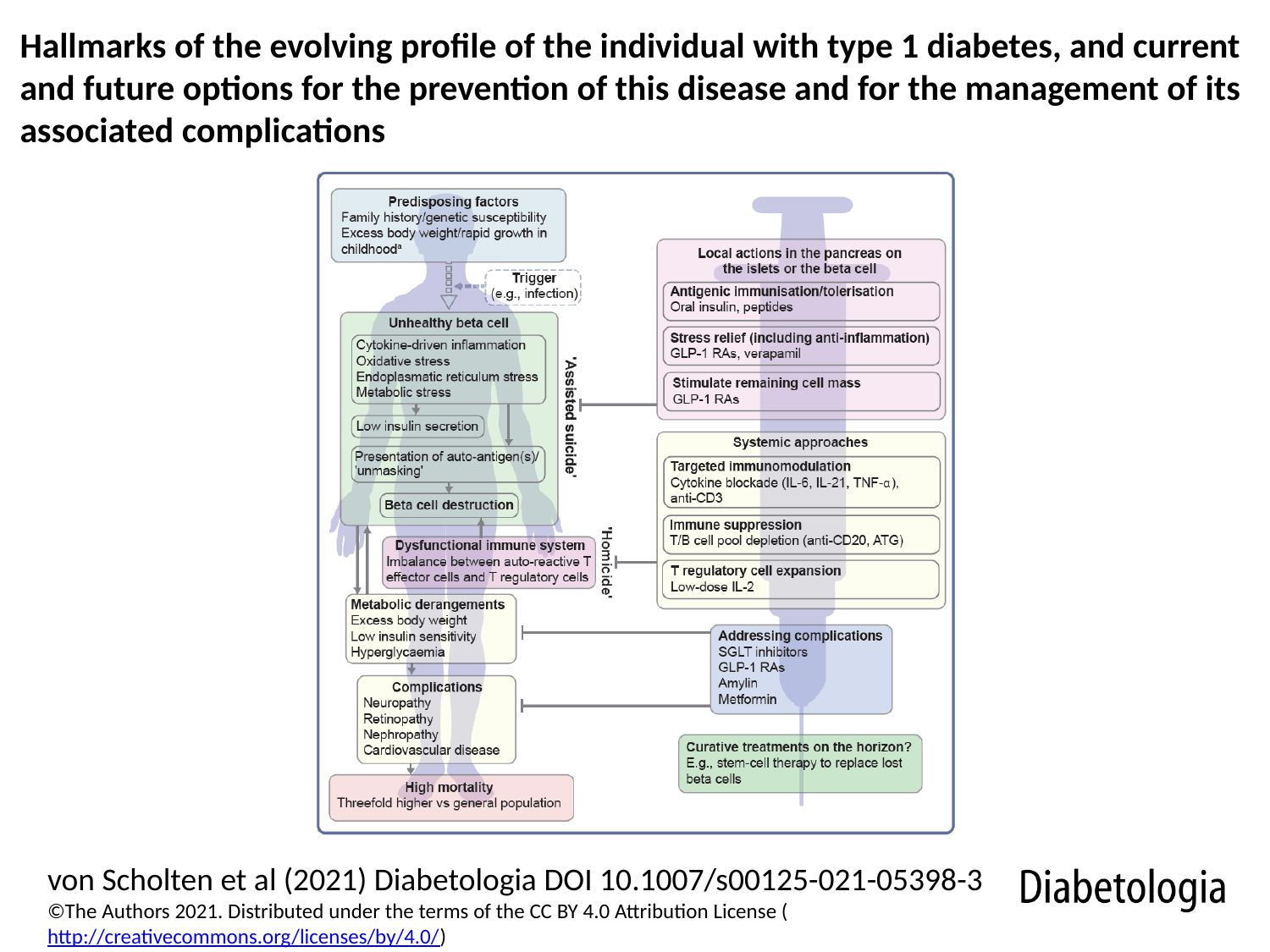

Hallmarks of the evolving profile of the individual with type 1 diabetes, and current and future options for the prevention of this disease and for the management of its associated complications
von Scholten et al (2021) Diabetologia DOI 10.1007/s00125-021-05398-3
©The Authors 2021. Distributed under the terms of the CC BY 4.0 Attribution License (http://creativecommons.org/licenses/by/4.0/)
